# The relationship between sport types, sex and visual attention as assessed in a multiple object tracking task

**DOI:** 10.3389/fpsyg.2023.1099254

**Published:** 2023-02-23

**Authors:** Peng Jin, Zi-Qi Zhao, Xiao-Feng Zhu

**Affiliations:** ^1^Department of Physical Education, Southeast University, Nanjing, China; ^2^Southeast University Research Institute of Sports Science, Nanjing, China; ^3^School of Elite Sport, Shanghai University of Sport, Shanghai, China

**Keywords:** open skill sport, closed skill sport, multiple object tracking, visual attention, sex difference

## Abstract

This study was conducted to examine differences in visual attention according to sports type and sex. In total, 132 participants [open-skill sport athletes (basketball players), closed-skill sport athletes (swimmers), and non-athletes; *n* = 22 men and 22 women each] aged 19–24 years performed a multiple object tracking (MOT) task, which is a well-established paradigm for the assessment of visual attention. Visual tracking accuracy was affected by the sport type (*p* < 0.001), being superior among basketball players than among swimmers and non-athletes, with no significant difference between the latter groups. It also varied by sex (*p* < 0.001), being superior among males than among females. Significant interaction between the sport type and sex was observed (*p* < 0.001), with male and female basketball players showing similar tracking accuracy. Our results demonstrate that open-skill sport activities strongly related to visual attention, as estimated by MOT task performance, and that sex plays a role in this performance. They also indicate that females might gain a greater visual attention advantage from open than from closed-skill sports participation, as long-term open-skill sports training appeared to minimize the sex difference in visual attention.

## Introduction

Visual attention has perhaps been the most active area of study in perceptual-cognitive research in the last several decades (Harris et al., 2020). Visual attention is widely accepted in psychology and can be described as the cognitive system’s selection of a subset of relevant information for further processing (Meyerhoff and Papenmeier, 2020). It plays an important role in most activities of daily living, including assessments of traffic, work, and sports (Hüttermann and Memmert, 2017). In sports, athletes must continuously pay attention to the environment to execute timely and correct decisions (Walsh, 2014). This skill is especially critical in sports such as basketball, in which players are required to track the ball in dynamically changing and unpredictable situations while simultaneously monitoring the position of other players, including teammates and opponents on the court (Gallotta et al., 2020; Legault et al., 2022). Thus, the investigation of visual attention in a sports environment may provide a deeper understanding of the involved cognitive mechanisms to inform better performance of many real-life tasks.

Several studies have examined the relationship of sports participation to visual attention, and have yielded somewhat conflicting results; sports training has been found to have positive transfer effects on visual attention (Faubert, 2013; Zhang et al., 2021), whereas other studies do not find this (Memmert et al., 2009). Some of these discrepant findings may be because different types of sports activities may exert differential influences on visual attention (Gallotta et al., 2020). Thus, increasingly more investigations have focused on the relationship between the type of sports training and visual attention. Sports may be categorized into two types based on their cognitive demands: open skill sports and closed skill sports (Poulton, 1957; Ke et al., 2021). In open skill sports (e.g., basketball, tennis, squash, or boxing), participants are required to react and adapt in an unpredictable and dynamically changing environment. By contrast, closed skill sports (e.g., swimming, running, or cycling) are performed in an environment that is internally paced, predictable, and highly consistent (Gu et al., 2019; Coyne et al., 2021; Koch and Krenn, 2021). Regarding the different characteristic by type of sport, researchers have found that athletes in open skill sports show better performance in cognitive function (Wang et al., 2013), visuospatial attention (Tsai et al., 2016), and other cognitive tasks than have closed-skill sports athletes (Voss et al., 2010). Despite these clear sport type-related differences in cognitive abilities, however, evidence indicating which types of sport activity are associated with superior visual attention performance remains insufficient.

Sex differences in visual attention have also attracted research interest. Some studies have investigated sex differences in visual attention among the general population. For example, Roudaia and Faubert (2017) found that males showed superior visual attention compare with females in a visual tracking task, whereas Merritt et al. (2007) found no sex difference in visual selection task performance. Only a few studies have explored sex differences in visual attention in athletes, highlighting the demand for more study in this area. One study indicated that male athletes show superior cognition compare with female athletes (Lum et al., 2002), and Legault et al. (2022) reported a significant sex difference in visual tracking performance favoring male athletes. By contrast, another study reported no superior performance between male and female volleyball players on a visual selective attention task (Alves et al., 2013). Similarly, another study found no statistically significant sex difference on a visual spatial attention task among volleyball players (Notarnicola et al., 2014b). Thus, the existence of a sex difference in athletes’ visual attention remains controversial; previous findings should be further confirmed and more investigation in this area is needed.

The multiple object tracking (MOT) task is a well-established paradigm used for investigating visual attention in laboratory research (Pylyshyn and Storm, 1988). In a classic MOT task, target and distractor objects are presented on a screen. A subset of the objects are cued as being target objects (typically by their changing color or flashing) for a short period of time. Thereafter, all objects move around on the screen for several seconds, and at the end of a given trail the observer is asked to identify the original target objects (Meyerhoff et al., 2017). The MOT task is considered to be one of the most popular and powerful tools for exploring the underlying mechanisms involved in visual attention, and the best method for the assessment of distributed, selective, and sustained attention skills (Qiu et al., 2018). Some previous studies demonstrating an expertise effect on performance in Mot task. In domains as varied as radar operator expertise (Allen et al., 2004), video-game expertise (Green and Bavelier, 2006), and sport expertise (Martín et al., 2017; Jin et al., 2020), all of these studies have used the MOT task to display that expertise in visual attention provides a greater advantages. In addition, the MOT task paradigm is similar to the requirements of an open skill sports situation (Mangine et al., 2014). In some open skill sports that have high cognitive demands, such as basketball, players have to react within continuously changing conditions. They need to concurrently focus not only on the ball and the field but also on the movement and position of opponents and teammates (Zarić et al., 2018). In contrast, closed skill sports, such as swimming, are performed in a predictable and stable environments (Koch and Krenn, 2021) that require less such cognitive activities and attention demands (Cooper et al., 2018; Gallotta et al., 2020). Therefore, it is reasonable to postulate that open skill sports athletes may exhibit performance on the MOT task superior to closed skill sports athletes owing to the nature of the type of sport. However, this remains controversial. For example, one study from Memmert et al. (2009) found that handball athletes show no better tracking accuracy on the MOT task than either track athletes or non-athletes. Thus, whether MOT task performance can be improved by engaging in a given type of sport remains unclear. In addition, there is a limited number of studies about sex differences in MOT task performance, in general and according to sports type (open-vs. closed-skill).

Thus, this study was conducted to examine whether there are differences between types of sport and between men and women in a visual attention task. The two main objectives were as follows: (1) to investigate possible differences in performance measures on the MOT task among athletes trained in an open skill sport, athletes trained in a closed skill sport, and non-athletes; and (2) to assess whether there is a sex difference in MOT task performance. Our hypotheses were twofold. The first hypothesis was that basketball athletes would exhibit better tracking accuracy performance on the MOT task than both athletes trained in swimming and non-athletes. Based on the literature we mentioned above (Alves et al., 2013; Notarnicola et al., 2014a), our second hypothesis was that sex differences for MOT task performance would be revealed only in non-athletes and swimmers, not in the basketball players.

## Materials and methods

### Participants

Sample size calculations were computed by analysis of variance (ANOVA) *F*-test and performed using G*Power 3.1.9.2 software. Using a medium effect size of 0.25, an alpha level of 0.05, and power of 0.80 (Cohen, 1992), the result indicated that a sample size of at least 158 participants was required. However, the number of participants in present study was slightly below the limit due to COVID-19 related restrictions at the time. Therefore, the final total sample encompassed 132 participants in our study. We recruited 44 open-skill sport athletes, 44 closed-skill sport athletes, and 44 non-athletes (22 men and 22 women each). The open skill sport athletes were recruited from eight China University Basketball Association (CUBA) teams. These athletes comprised 22 men (mean age: 21.62 ± 2.07 years) with a mean of 9.46 (SD = 2.92) years of basketball training experience, and a mean of 12.36 (SD = 2.14) training hours per week as well as 22 women (mean age: 21.48 ± 1.92 years), with a mean of 9.17 (SD = 2.44) years of basketball training experience and a mean of 12.42 (SD = 2.37) training hours per week. All players were first-level national athletes. The closed skill sport athletes were com-posed of 22 men (mean age: 21.89 ± 2.32 years) with a mean of 10.05 (SD = 2.63) years of swim training experience, and a mean of 12.87 (SD = 1.82) training hours per week as well as 22 women (mean age: 21.36 ± 1.58 years), with a mean of 9.78 (SD = 1.53) years of swim training experience and a mean of 12.62 (SD = 2.37) training hours per week. All of these athletes were also first-level national athletes. The non-athlete group was composed of 22 men (mean age: 21.17 ± 1.28 years) and 22 women (mean age: 21.05 ± 1.41 years) who were college students and had never participated in open skill sports training or any other sports. All participants reported normal or corrected-to-normal levels of visual function. All participants received a verbal explanation of the research and experimental procedures. The study protocol was approved by the Ethics Committee of Shanghai University of Sport (No. 2015003SUS). All participants provided written informed consent prior to the start of the experiment. Each participant who completed the study received a small monetary compensation for their time.

### Stimuli, apparatus, and procedure

**Figure 1:**
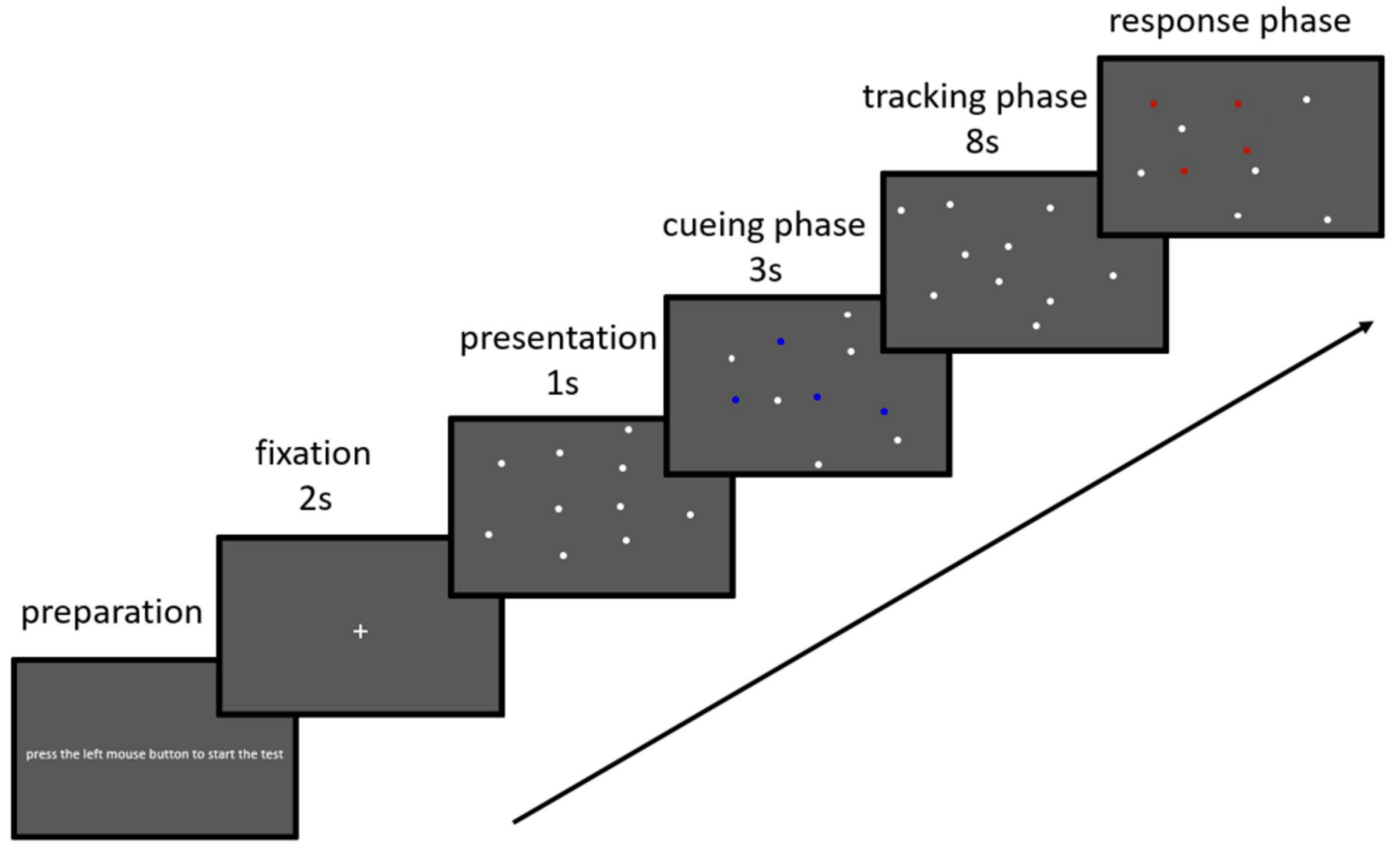
Schematic diagram of the visual stimuli presented in one trial of the multiple object tracking task. Ten filled white circles are presented on the screen. In each trial, four of the circles turn blue and flicker for 3 s before turning back to white. The blue flickering circles indicate the targets for that trial. All 10 circles then move 10°/s for an 8-s tracking period. When the circles stop moving, the participants use a mouse to click over their choices for the target circles.

The experiment was conducted on a ThinkPad (ThinkBook Plus 17) laptop running Windows 10. Visual stimuli were created using MATLAB R2016a (MathWorks, Natick, MA) and Psychtoolbox 3.0 software. Stimuli were presented on a 17-inch monitor with a resolution of 3,072 × 1,440 pixels and a refresh rate of 120 Hz. Individual participants were seated ~ 55 cm in front of the monitor of the laptop and were tested individually in a quiet room. The entire experiment consisted of 30 trials in two blocks separated by a 3-min rest period (total, ~ 12 min). Before the start of the formal session, the participants completed six practice trials to ensure that they were familiar with the procedure. At the beginning of each test block, the instruction “press the left mouse button to start the task” was presented on the screen. In each trial, a white fixation symbol (+) was displayed in the center of a gray background (visual field, 37.98 × 21.0°) for 2,000 ms, followed by the presentation of 10 white-field circles (0.65 diameter) for 1,000 ms. Four filled circles were highlighted blue and flickered three times for a total of 3 s to mark them as the targets. Thereafter, the target circles returned to white so that no cue remained to discriminate them from the untracked items (distractors). Next, the 10 filled circles moved in random directions at a constant speed of 10°/s, with the movement of each circle affected only by collisions (The dots changed their directions randomly when they reached the edge of the screen border. There were no extra constraints in the dots’ trajectories hence there was the possibility that they crossing each other for an instant). After 8 s, the filled circles stopped moving. The participants were instructed to identify the targets by pressing a mouse button (Figure 1). Their responses also triggered the start of the next trial. The tracking accuracy recorded with all other study data in MATLAB R2016a (The Math Works).

### Statistical analysis

The statistical analyses were performed using SPSS 23.0. A Univariate analysis of variance (UNIANOVA) was constructed with sex (male and female) and group (basketball players, swimmers, and college students) serving as independent variables and MOT task tracking accuracy serving as the dependent variable. The simple effects test was performed to identify any significant interaction and simple comparisons followed by Bonferroni correction was used. Effect sizes (Cohen’s *f*) were calculated; *d* = 0.10 was considered to represent a small effect, *d* = 0.25 was considered to represent a medium’ effect, and *d* = 0.40 was considered to represent a large effect *p* < 0.05 was considered to be significant. The tracking accuracy was calculated by determining the percentage of correctly selected targets across all experimental times for each participant. For example, if a participant identified all four targets 15 out of 30 times, the tracking accuracy was 50%.

## Results

**Figure 2:**
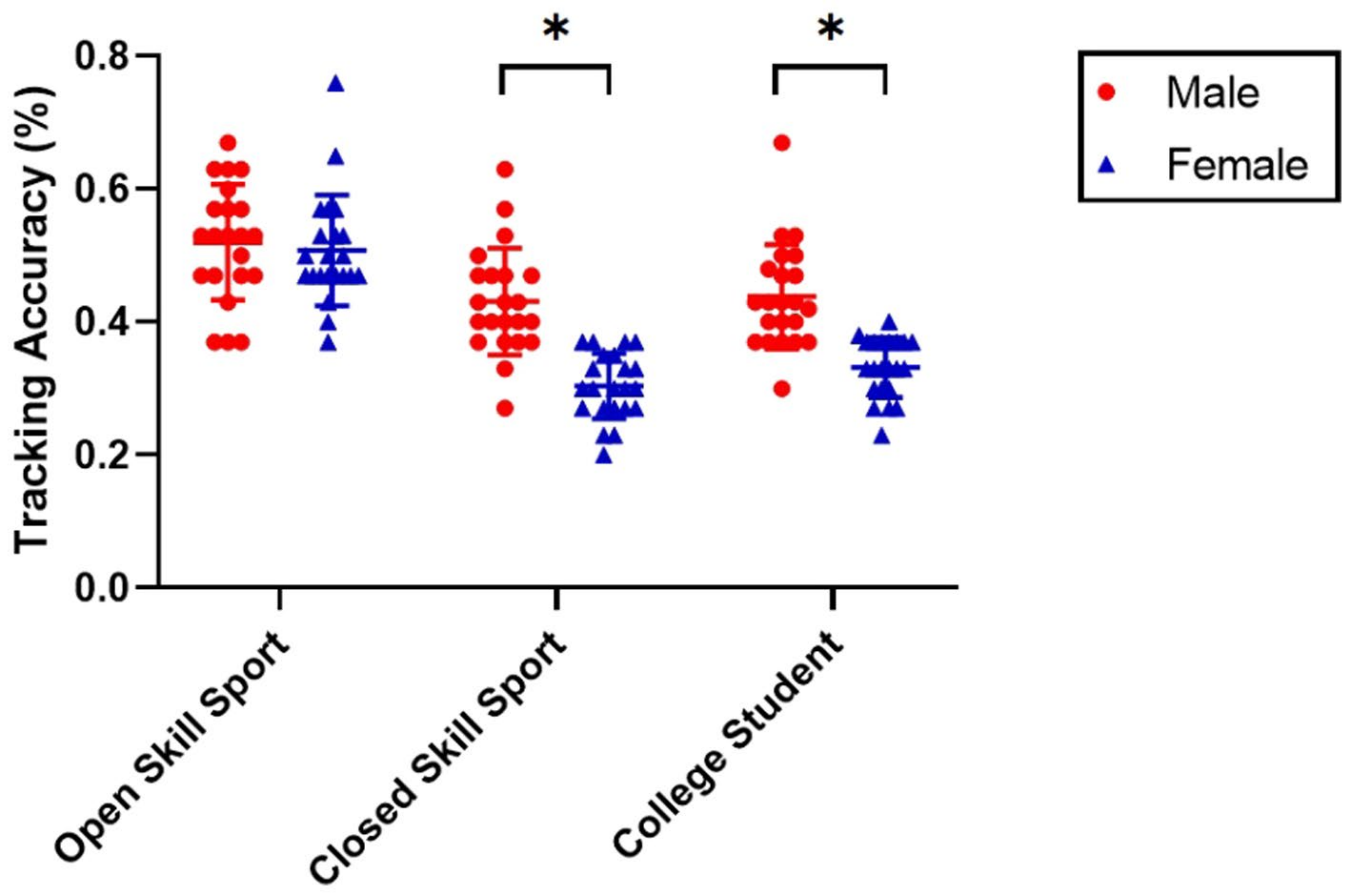
Comparison of the tracking accuracy between groups. *p<0.05.

The means and standard deviations of the tracking accuracy for the groups of open skill sport and closed skill sport or college student of both sex are shown in Figure 2. For the dependent variable of tracking accuracy, our UNIANOVA results indicated significant main effects for sex, *F*(1,126) = 42.329, *p* < 0.001, *η*_P_^2^ = 0.251 and for group, *F*(2,126) = 53.643, *p* < 0.001, *η*_P_^2^ = 0.460 (see Table 1). Importantly, a significant interaction was also observed between sex and group, *F*(2,126) = 7.874, *p* < 0.001, *η*_P_^2^ = 0.111.

**Table 1:** Univariate analysis of variance (UNIANOVA) results for between-subjects effects.

Source	SoS-III	*df*	Mean square	*F*	*p*	*η*_P_^2^
Corrected model	0.869^a^	5	0.174	33.073	<0.001	0.568
Intercept	23.504	1	23.504	4470.385	<0.001	0.973
Sex	0.223	1	0.223	42.329	<0.001	0.251
Group	0.564	2	0.282	53.643	<0.001	0.46
Sex × group	0.083	2	0.041	7.874	0.001	0.111
Error	0.662	126	0.005			
Total	25.036	132				
Corrected total	1.532	131				

Dependent variable = tracking accuracy. ^a^*R*2 = 0.568 (adjusted *R*2 = 0.550). SoS-III, type III sum of squares; *df*, degrees of freedom.

Further simple effects analyses showed that for sex, male basketball athletes (*M* = 0.52, SD = 0.09) had significantly better tracking accuracy than male swimming athletes (*M* = 0.44, SD = 0.08; *p* < 0.01, *d* = 0.94) and then male college students (*M* = 0.43, SD = 0.08; *p* < 0.01, *d* = 1.06). However, the difference in tracking accuracy between male swimming athletes and college students failed to reach statistical significance (*p* = 0.26). An analysis by group revealed that male swimmers (*M* = 0.44, SD = 0.08) had significantly better tracking accuracy than female swimmers (*M* = 0.33, SD = 0.04; *p* < 0.01, *d* = 1.73); similarly, the tracking accuracy among male college students (*M* = 0.43, SD = 0.08) was significantly higher than among female college students (*M* = 0.30, SD = 0.05; *p* < 0.01, *d* = 1.94). By contrast, there was no significant difference in tracking accuracy between male basketball athletes (*M* = 0.52, SD = 0.09) and female basketball athletes (*M* = 0.50, SD = 0.08; see Table 2; Figure 2).

**Table 2:** Sex and group interactions.

Group	Sex	Mean difference	SE	*p* ^a^	95% CI	
					Lower limit	Upper limit
Open skill sport	Male vs. Female	0.012	0.022	0.576	−0.031	0.056
Closed skill sport	Male vs. Female	0.127*	0.022	<0.001	0.084	0.171
College student	Male vs. Female	0.107*	0.022	<0.001	0.064	0.15

CI = confidence interval; SE, standard error.

Dependent variable = tracking accuracy. ^*^The mean difference is significant at the.05 level. ^a^Adjustment for multiple comparisons (least significant difference test, equivalent to no adjustments).

## Discussion

The purpose of this study was to determine whether visual attention as assessed in the MOT task by tracking accuracy performance varied by type of sport (closed skill vs. open skill) or by sex among first-level national athletes. The results provided evidence to support our hypothesis that open skill athletes (in this case, basketball players) exhibit performance on the MOT task superior to closed skill athletes (swimmers) and to non-athletes (non-athletic college students). However, there was no significant difference in performance on the MOT task between swimmers and non-athletes. The results of this study were also concordant with our hypothesis regarding sex in that significant sex differences in tracking accuracy were observed for both the closed skill group and the non-athlete group. As expected, in the open skill sport group, we did not detect a difference between male basketball and female basketball players on tracking accuracy on the MOT task, a finding that has been little reported.

Regarding the differences by type of sport, we found that sport type is closely linked to visual attention, with tracking accuracies on the MOT task among basketball athletes being markedly better than those in both the closed skill sport group and the non-athlete group. A plausible explanation for this finding is that open skill athletes have to monitor numerous changing stimuli and continuously shifting positions to excel in their sport (Ong, 2017; Gökçe et al., 2021). In basketball, players have to allocate visual attention to track the targets of the ball, teammates, and opponents while inhibiting irrelevant information over time on the court. These tracking processes are similar to those used in the MOT task. In comparison, because closed skill sports take place in a predictable and relatively stable environment, there are fewer cognitive activities required and fewer demands on visual attention (Gu et al., 2019). Current evidence suggests that an enriched environment, such as basketball training, has positive effects on the brain (Qiu et al., 2019). Therefore, it is reasonable to suggest that the superior performance of the basketball players on tracking accuracy in the MOT task indicates that open skill sports activities may improve visual attention. This conclusion aligns with recent trends in research showing that open skill sports athletes have better cognitive performance than closed skill sports athletes and non-athletes (Mohammadi et al., 2016; Holfelder et al., 2020).

We found no difference in the tracking accuracies between swimming athletes and non-athletes, suggesting that closed skill sports with low cognitive demands, such as aerobic exercise or resistance training, yields no greater abilities in visual attention (Diamond and Ling, 2019). The disagreement between our results and the previous finding that the sport type is unrelated to MOT task performance differences among handball players, track athletes, and non-athletes (Memmert et al., 2009) may be related to the MOT task structure. In the previous study, the MOT task had a lesser tracking load, with 7 objects (3 targets and 4 distractors) used instead of the 10 objects used in our study, which may not have provided sufficient sensitivity to detect a difference. It is also the reason why the tracking accuracy is low in comparison to other studies. Another explanation is that the object speed, in this study, the object speed is 10°/S, which is faster than other studies (Zhang et al., 2021), the previous research showed that tracking accuracy declines as object speed increases in the MOT task (Ma and Flombaum, 2013). Taken together, our findings extended those of previous studies and were consistent with our hypothesis that open skill sports athletes would exhibit visual attention as assessed in the MOT task superior to that of closed skill sports athletes.

The present study also found that the differences of tracking performance between male and female in the MOT task, with males demonstrating tracking accuracy superior to that of females in experimental groups. Our finding is consistent with existing research assessing visual attention on a MOT task (Roudaia and Faubert, 2017). Other studies using visual–spatial tasks (Notarnicola et al., 2014b), Silverman et al. (2007) have also shown that males demonstrate superior performance compared with females. This finding may be attributable to asymmetry in brain structures and the percentages of white vs. gray matter (Gur and Gur, 2017). Apart from potential biological factors, a difference in opportunities to participate in electronic video games and sports in daily life could result in the sex effects observed in the MOT task (Green et al., 2010). Another reason may be sociocultural environments; some findings have demonstrated a relationship between sex differences and national indices of gender equality for sustained attention (Riley et al., 2016; Roudaia and Faubert, 2017). Our findings thus extend previous research and further support the view that sex differences exist in visual attention.

We also found an interaction between group (open vs. closed skill sports athletes vs. non-athletes) and sex in the present study. A statistically significant sex difference was observed in tracking accuracy on the MOT task for the closed skill sport group (swimmers), whereas male basketball players did not show tracking accuracy performance superior to female basketball players (open skill sport). As far as we know, only a few studies have shown an absence of sex differences in visual attention abilities among open skill sports athletes. One recent study demonstrated that male basketball players did not exhibit better tracking accuracy than women basketball players on the MOT task (Jin and Fan, 2022). Two other studies also drew the same conclusion although they used different designs to assess visual attention in open skill sports athletes. One of those studies reported that athletes of an open skill sport (volleyball) showed no sex differences in the performance of a visual–spatial task (Notarnicola et al., 2014b). The other study also failed to find a statistically significant difference between female and male volleyball athletes in visual selective attention (Alves et al., 2013). A plausible explanation for these findings is that the athletes were participating in sports in which the environmental conditions for both men and women required similar perceptual skills (Mc Leod, 1987). Another possible explanation is that changes in hormone levels among women undergoing such sports training appears to get a higher advantage in related cognition function. Sex hormones have been shown to have a strong impact on performance in attention tasks (Holländer et al., 2005). Another study (Lord and Garrison, 1998) found that female basketball athletes who showed a greater benefit compare with female swimming and track athletes in spatial abilities had increased androgen levels. These findings could explain why sex differences only existed in the closed skill sports group, not in the open skill sports group.

The current study is the first, to our knowledge, to show that the sport type and sex affect visual tracking (MOT task) performance. However, some limitations must be taken into consideration. The main shortcoming was that the participating basketball players were not representative of the whole open-skill sport population. We recommend that future research be performed with athletes in a wider range of open-skill sports, in particular individual sports such as tennis and boxing. Furthermore, it is a cross-sectional design, not a Randomized Controlled trial. It is impossible for our study design to assign participants randomly, it is always possible that females need not necessarily gain advantage from open sport; maybe only those females with better MOT were able to remain in the sport over the years. Therefore, a longitudinal studies are needed to observe changes in open-and closed-skill athletes’ visual attention abilities over time in the future study. Lastly, the small sample size limits the generalizability of our study. We did not reach the sample size of 158 participants we expected, for the COVID-19 pandemic have made it difficult to recruit a larger number of participants, future study design will consider increasing the sample size to increasing the generalizability and validity of the results.

In conclusion, the current study demonstrates that open-skill sports athletes have visual tracking accuracy superior to that of closed-skill sport athletes and non-athletic college students on MOT task. This finding indicates that the cognitive requirements of the sports environment provided by open skill sports may transfer to enhanced visual attention abilities. The present study also revealed a sex difference in visual attention among non-athletes and among athletes in a closed skills sport, highlighting the need to control for sex in research comparing attentional tracking abilities across different participant groups. Additionally, no difference in tracking accuracy on the MOT task was found between female and male basketball athletes, supporting the idea that training in open skill sports may reduce sex differences in visual attention. These findings highlighting the necessary to control for sex in researches comparing visual tracking abilities in different participant groups, and also give sport psychology practitioners and coaches clues to be used in identifying athletes who might need to further improve this capacity.

## Data availability statement

The original contributions presented in the study are included in the article/Supplementary material, further inquiries can be directed to the corresponding author.

## Ethics statement

The studies involving human participants were reviewed and approved by Ethics Committee of Shanghai University of Sport. The patients/participants provided their written informed consent to participate in this study.

## Author contributions

PJ and Z-QZ: conceptualization. X-FZ: methodology and supervision. PJ: writing—original draft. PJ and X–FZ: writing—review and editing. All authors contributed to the article and approved the submitted version.

## Conflict of interest

The authors declare that the research was conducted in the absence of any commercial or financial relationships that could be construed as a potential conflict of interest.

## Publisher’s note

All claims expressed in this article are solely those of the authors and do not necessarily represent those of their affiliated organizations, or those of the publisher, the editors and the reviewers. Any product that may be evaluated in this article, or claim that may be made by its manufacturer, is not guaranteed or endorsed by the publisher.

## References

[ref1] Allen R. Mcgeorge P. Pearson D. Milne A. B. (2004). Attention and expertise in multiple target tracking. Appl. Cogn. Psychol. 18, 337–347. doi: 10.1002/acp.975

[ref2] Alves H. Voss M. W. Boot W. R. Deslandes A. Cossich V. Salles J. I.. (2013). Perceptual-cognitive expertise in elite volleyball players. Front. Psychol. 4:36. doi: 10.3389/fpsyg.2013.00036, PMID: 23471100 PMC3590639

[ref3] Cooper S. B. Dring K. J. Morris J. G. Sunderland C. Bandelow S. Nevill M. E. (2018). High intensity intermittent games-based activity and adolescents’ cognition: moderating effect of physical fitness. BMC Public Health 18, 1–14. doi: 10.1186/s12889-018-5514-6, PMID: 29739386 PMC5941716

[ref01] Cohen J. (1992). Statistical power analysis. Curr. Dir. Psychol. Sci. 1, 98–101. doi: 10.1111/1467-8721.ep10768783, PMID: 23378899

[ref4] Coyne J. O. Coutts A. J. Newton R. U. Haff G. G. (2021). The influence of mental fatigue on sessional ratings of perceived exertion in elite open and closed skill sports athletes. J. Strength Cond. Res. 35, 963–969. doi: 10.1519/JSC.0000000000003980, PMID: 33752221

[ref5] Diamond A. Ling D. S. (2019). Aerobic-exercise and resistance-training interventions have been among the least effective ways to improve executive functions of any method tried thus far. Dev. Cogn. Neurosci. 37:100572. doi: 10.1016/j.dcn.2018.05.001, PMID: 29909061 PMC6969311

[ref6] Faubert J. (2013). Professional athletes have extraordinary skills for rapidly learning complex and neutral dynamic visual scenes. Sci. Rep. 3, 1–3. doi: 10.1038/srep01154, PMID: 23378899 PMC3560394

[ref7] Gallotta M. C. Bonavolontà V. Zimatore G. Iazzoni S. Guidetti L. Baldari C. (2020). Effects of open (racket) and closed (running) skill sports practice on Children’s attentional performance. Open Sports Sci. J. 13, 105–113. doi: 10.2174/1875399X02013010105

[ref8] Gökçe E. Güneş E. Arı F. Hayme S. Nalçacı E. (2021). Comparison of the effects of open-and closed-skill exercise on cognition and peripheral proteins: a cross-sectional study. PLoS One 16:e0251907. doi: 10.1371/journal.pone.0251907, PMID: 34086693 PMC8177547

[ref9] Green C. S. Bavelier D. (2006). Enumeration versus multiple object tracking: the case of action video game players. Cognition 101, 217–245. doi: 10.1016/j.cognition.2005.10.004, PMID: 16359652 PMC2896820

[ref10] Green C. S. Li R. Bavelier D. (2010). Perceptual learning during action video game playing. Top. Cogn. Sci. 2, 202–216. doi: 10.1111/j.1756-8765.2009.01054.x 25163784

[ref11] Gu Q. Zou L. Loprinzi P. Quan M. Huang T. (2019). Effects of open versus closed skill exercise on cognitive function: a systematic review. Front. Psychol. 10:1707. doi: 10.3389/fpsyg.2019.01707, PMID: 31507472 PMC6718477

[ref12] Gur R. C. Gur R. E. (2017). Complementarity of sex differences in brain and behavior: from laterality to multimodal neuroimaging. J. Neurosci. Res. 95, 189–199. doi: 10.1002/jnr.23830, PMID: 27870413 PMC5129843

[ref13] Harris D. J. Wilson M. R. Crowe E. M. Vine S. J. (2020). Examining the roles of working memory and visual attention in multiple object tracking expertise. Cogn. Process. 21, 209–222. doi: 10.1007/s10339-020-00954-y, PMID: 32016685 PMC7203592

[ref14] Holfelder B. Klotzbier T. J. Eisele M. Schott N. (2020). Hot and cool executive function in elite-and amateur-adolescent athletes from open and closed skills sports. Front. Psychol. 11:694. doi: 10.3389/fpsyg.2020.00694, PMID: 32373029 PMC7177013

[ref15] Holländer A. Hausmann M. Hamm J. P. Corballis M. C. (2005). Sex hormonal modulation of hemispheric asymmetries in the attentional blink. J. Int. Neuropsychol. Soc. 11, 263–272. doi: 10.1017/S1355617705050319, PMID: 15892902

[ref16] Hüttermann S. Memmert D. (2017). The attention window: a narrative review of limitations and opportunities influencing the focus of attention. Res. Q. Exerc. Sport 88, 169–183. doi: 10.1080/02701367.2017.1293228, PMID: 28332919

[ref17] Jin P. Fan M. T. (2022). Team ball sport experience minimizes sex difference in visual attention. Front. Psychol. 13:987672. doi: 10.3389/fpsyg.2022.987672, PMID: 36312175 PMC9606817

[ref18] Jin P. Li X. Ma B. Guo H. Zhang Z. Mao L. (2020). Dynamic visual attention characteristics and their relationship to match performance in skilled basketball players. PeerJ 8:e9803. doi: 10.7717/peerj.9803, PMID: 32879809 PMC7443082

[ref19] Ke L. Lanlan Z. Jian Z. Jianing W. (2021). Comparison of open-skill and closed-skill exercises in improving the response inhibitory ability of the elderly: a protocol for a randomised controlled clinical trial. BMJ Open 11:e051966. doi: 10.1136/bmjopen-2021-051966, PMID: 34815282 PMC8611442

[ref20] Koch P. Krenn B. (2021). Executive functions in elite athletes–comparing open-skill and closed-skill sports and considering the role of athletes' past involvement in both sport categories. Psychol. Sport Exerc. 55:101925. doi: 10.1016/j.psychsport.2021.101925

[ref21] Legault I. Sutterlin-Guindon D. Faubert J. (2022). Perceptual cognitive abilities in young athletes: a gender comparison. PLoS One 17:e0273607. doi: 10.1371/journal.pone.0273607, PMID: 36044462 PMC9432702

[ref22] Lord T. R. Garrison J. (1998). Comparing spatial abilities of collegiate athletes in different sports. Percept. Mot. Skills 86, 1016–1018. doi: 10.2466/pms.1998.86.3.101, PMID: 9656301

[ref23] Lum J. Enns J. T. Pratt J. (2002). Visual orienting in college athletes: explorations of athlete type and gender. Res. Q. Exerc. Sport 73, 156–167. doi: 10.1080/02701367.2002.10609004, PMID: 12092890

[ref24] Ma Z. Flombaum J. I. (2013). Off to a bad start: uncertainty about the number of targets at the onset of multiple object tracking. J. Exp. Psychol. Hum. Percept. Perform. 39, 1421–1432. doi: 10.1037/a0031353, PMID: 23339351

[ref25] Mangine G. T. Hoffman J. R. Wells A. J. Gonzalez A. M. Rogowski J. P. Townsend J. R.. (2014). Visual tracking speed is related to basketball-specific measures of performance in NBA players. J. Strength Cond. Res. 28, 2406–2414. doi: 10.1519/JSC.0000000000000550, PMID: 24875429

[ref26] Martín A. Sfer A. M. D'Urso Villar M. A. Barraza J. F. (2017). Position affects performance in multiple-object tracking in rugby union players. Front. Psychol. 8:1494. doi: 10.3389/fpsyg.2017.01494, PMID: 28951725 PMC5599788

[ref27] Mc Leod B. (1987). Sex, structured sport activity, and measurement of field dependence. Percept. Mot. Skills 64, 452–454. doi: 10.2466/pms.1987.64.2.452

[ref28] Memmert D. Simons D. J. Grimme T. (2009). The relationship between visual attention and expertise in sports. Psychol. Sport Exerc. 10, 146–151. doi: 10.1016/j.psychsport.2008.06.002

[ref29] Merritt P. Hirshman E. Wharton W. Stangl B. Devlin J. Lenz A. (2007). Evidence for gender differences in visual selective attention. Personal. Individ. Differ. 43, 597–609. doi: 10.1016/j.paid.2007.01.016

[ref30] Meyerhoff H. S. Papenmeier F. (2020). Individual differences in visual attention: a short, reliable, open-source, and multilingual test of multiple object tracking in PsychoPy. Behav. Res. Methods 52, 2556–2566. doi: 10.3758/s13428-020-01413-4, PMID: 32495028

[ref31] Meyerhoff H. S. Papenmeier F. Huff M. (2017). Studying visual attention using the multiple object tracking paradigm: a tutorial review. Atten. Percept. Psychophysiol. 79, 1255–1274. doi: 10.3758/s13414-017-1338-1, PMID: 28584953

[ref32] Mohammadi N. Rostami R. Alborzi M. (2016). Visual skills of the female athletes in team and individual sports. Ann. Appl. Sport Sci. 4, 69–77. doi: 10.18869/acadpub.aassjournal.4.4.69

[ref33] Notarnicola A. Maccagnano G. Pesce V. Tafuri S. Novielli G. Moretti B. (2014a). Visual-spatial capacity: gender and sport differences in young volleyball and tennis athletes and non-athletes. BMC. Res. Notes 7:57. doi: 10.1186/1756-0500-7-57, PMID: 24447526 PMC3925789

[ref34] Notarnicola A. Maccagnano G. Pesce V. Tafuri S. Novielli G. Moretti B. (2014b). Visual-spatial capacity: gender and sport differences in young volleyball and tennis athletes and non-athletes. BMC. Res. Notes 7, 1–5. doi: 10.1186/1756-0500-7-57, PMID: 24447526 PMC3925789

[ref35] Ong N. (2017). Reactive stress tolerance in elite athletes: differences in gender, sport type, and competitive level. Cognitie, Creier, Comportament/Cognition, Brain, Behavior 21, 189–202. doi: 10.24193/cbb.2017.21.11

[ref36] Poulton E. (1957). On prediction in skilled movements. Psychol. Bull. 54, 467–478. doi: 10.1037/h0045515 13485273

[ref37] Pylyshyn Z. W. Storm R. W. (1988). Tracking multiple independent targets: evidence for a parallel tracking mechanism. Spat. Vis. 3, 179–197. doi: 10.1163/156856888X00122, PMID: 3153671

[ref38] Qiu F. Pi Y. Liu K. Li X. Zhang J. Wu Y. (2018). Influence of sports expertise level on attention in multiple object tracking. PeerJ 6:e5732. doi: 10.7717/peerj.5732, PMID: 30280051 PMC6166630

[ref39] Qiu F. Pi Y. Liu K. Zhu H. Li X. Zhang J.. (2019). Neural efficiency in basketball players is associated with bidirectional reductions in cortical activation and deactivation during multiple-object tracking task performance. Biol. Psychol. 144, 28–36. doi: 10.1016/j.biopsycho.2019.03.008, PMID: 30902565

[ref40] Riley E. Okabe H. Germine L. Wilmer J. Esterman M. DeGutis J. (2016). Gender differences in sustained attentional control relate to gender inequality across countries. PLoS One 11:e0165100. doi: 10.1371/journal.pone.0165100, PMID: 27802294 PMC5089545

[ref41] Roudaia E. Faubert J. (2017). Different effects of aging and gender on the temporal resolution in attentional tracking. J. Vis. 17:1. doi: 10.1167/17.11.1, PMID: 28862709

[ref42] Silverman I. Choi J. Peters M. (2007). The hunter-gatherer theory of sex differences in spatial abilities: data from 40 countries. Arch. Sex. Behav. 36, 261–268. doi: 10.1007/s10508-006-9168-6, PMID: 17351740

[ref43] Tsai C.-L. Wang C.-H. Pan C.-Y. Chen F.-C. Huang S.-Y. Tseng Y.-T. (2016). The effects of different exercise types on visuospatial attention in the elderly. Psychol. Sport Exerc. 26, 130–138. doi: 10.1016/j.psychsport.2016.06.013

[ref44] Voss M. W. Kramer A. F. Basak C. Prakash R. S. Roberts B. (2010). Are expert athletes ‘expert’in the cognitive laboratory? A meta-analytic review of cognition and sport expertise. Appl. Cogn. Psychol. 24, 812–826. doi: 10.1002/acp.1588

[ref45] Walsh V. (2014). Is sport the brain’s biggest challenge? Curr. Biol. 24, R859–R860. doi: 10.1016/j.cub.2014.08.003, PMID: 25247362

[ref46] Wang C.-H. Chang C.-C. Liang Y.-M. Shih C.-M. Chiu W.-S. Tseng P.. (2013). Open vs. closed skill sports and the modulation of inhibitory control. PLoS One 8:e55773. doi: 10.1371/journal.pone.0055773, PMID: 23418458 PMC3572130

[ref47] Zarić I. Dopsaj M. Marković M. (2018). Match performance in young female basketball players: relationship with laboratory and field tests. Int. J. Perform. Anal. Sport 18, 90–103. doi: 10.1080/24748668.2018.1452109

[ref48] Zhang Y. Lu Y. Wang D. Zhou C. Xu C. (2021). Relationship between individual alpha peak frequency and attentional performance in a multiple object tracking task among ice-hockey players. PLoS One 16:e0251443. doi: 10.1371/journal.pone.0251443, PMID: 34043652 PMC8158945

